# Six-Year Outcomes of 25-Gauge Chandelier Illumination-Assisted Scleral Buckling

**DOI:** 10.1155/2021/4628160

**Published:** 2021-10-04

**Authors:** Hui Li, Conghui Zhang, Jiayi Wei, Khusbu Keyal, Fang Wang

**Affiliations:** Department of Ophthalmology, Shanghai Tenth People's Hospital, Affiliated to Tongji University, Shanghai 200072, China

## Abstract

**Objectives:**

To report the long-term results of scleral buckling using 25-gauge chandelier illumination.

**Methods:**

The medical records of all patients presenting to Shanghai Tenth People's Hospital with simple rhegmatogenous retinal detachment (RRD) from June 2013 to Oct 2015 were retrospectively reviewed in this consecutive case series. All patients underwent preoperative and postoperative best corrected visual acuity (BCVA), B-ultrasound, fundus photography, and optical coherence tomography examination. Ultrasound biomicroscopy (UBM) was obtained postoperatively.

**Results:**

Ten patients (10 eyes) were included in the final analysis. Of 10 patients, the average age was 49.3 ± 18.9 years old, the average duration of RRD was 30.9 ± 53.3 days, and the mean follow-up period was 6.2 ± 0.9 years. There were nine eyes with myopia and four eyes with macular detachment. The primary anatomical success rate was 90%. Five eyes underwent 360-degree band with element surgery, and five eyes underwent element surgery alone. The average length of encircling band and element was 68.2 ± 1.3 mm and 10.5 ± 2.5 mm, respectively. There were no intraoperative or postoperative complications that occurred. The final BCVA was greater than or equal to 20/40 in nine eyes, of which four eyes achieved 20/20. UBM examination of the 25-gauge chandelier insertion site revealed no tissue proliferation.

**Conclusions:**

For simple rhegmatogenous retinal detachment treatment, 25-gauge chandelier illumination-assisted scleral buckling is a kind of effective and safe method.

## 1. Introduction

Rhegmatogenous retinal detachment (RRD) leads to anatomical abnormality of the retina and, consequently, visual function impairment. Scleral buckling, which was first introduced in the 1950s [1], is a classic surgery to treat RRD. Scleral buckling has unique advantages including faster visual rehabilitation, reducing the incidence of proliferative vitreoretinopathy, and decreasing the risk of cataract formation. However, due to the limitations of conventional scleral buckling and rapid development of vitrectomy technique and instrument, conventional scleral buckling is practiced and taught with declining trend and even faced a huge challenge of being eliminated. As early as 2013, United Kingdom National Ophthalmology Database reported 79.1% pars plana vitrectomy (PPV) and 12.1% scleral buckling was used in a total of 3403 eyes of primary RRD [2].

Conventional scleral buckling using indirect ophthalmoscope has some limitations, such as inverted image, limited surgical view, and surgical procedure cannot be shared and recorded [3]. In recent years, surgeons have made a lot of efforts like applying noncontact wide-angle viewing system, chandelier endoillumination [4], and 3D visualization system [5] to overcome the drawbacks of conventional scleral buckling. Scleral buckling under the microscope with chandelier endoillumination and noncontact wild-angle view system may reduce the chance of missing retinal breaks, make the peripheral retinal breaks or lesions more easily be identified and treated, and permit less extensive cryotherapy because of the improvement of illumination. In addition, the entire surgical procedure could be visualized and recorded, facilitating communication and teaching. Also, surgeons may feel more comfortable under the microscope than using indirect ophthalmoscopy.

Although there are many benefits, chandelier endoillumination-assisted scleral buckling has not been widely accepted in clinical practice. The main reason is that conventional scleral buckling is an extraocular procedure, but the chandelier endoillumination needs to be inserted into the vitreous cavity, which may increase potential risks such as infectious endophthalmitis, lens damage, vitreous incarceration or tissue proliferation, and phototoxicity [4]. Imai et al. [6] reported two cases of chandelier illumination-related complications. One patient experienced a new retinal break as the chandelier was removed from the cannula; another patient had lens touch by the tip of the endoilluminating chandelier during cryopexy. Sakono et al. [7] reported a case of bacterial endophthalmitis that developed three days after scleral buckling surgery with noncontact wide-angle viewing system and chandelier endoillumination. However, the long-term outcomes of scleral buckling using chandelier endoillumination has not been assessed. In this study, we aim to describe six-year results of 25-gauge chandelier endoillumination and noncontact wide-angle viewing system-assisted scleral buckling surgery.

## 2. Material and Methods

### 2.1. Experiment Methods

This study was a retrospective, nonrandomized, consecutive case series.

### 2.2. Data Collection

The medical records of patients who underwent scleral buckling surgery using 25-gauge chandelier illumination combined with noncontact wide-angle viewing system (Carl Zeiss Meditec Inc., Jena, Germany) of simple RRD at Shanghai Tenth People's Hospital between June 2013 and Oct 2015 were reviewed. All patients underwent pre- and postoperative best corrected visual acuity (BCVA), intraocular pressure (IOP), and slit lamp biomicroscopy examination. Other auxiliary test collected included fundus photography (Optos Inc., Marlborough, MA, USA), B-ultrasound (Aviso, Quantel Médical, Clermont-Ferrand, France), optical coherence tomography (OCT, Carl Zeiss Meditec Inc., Jena, Germany), and ultrasound biomicroscope (UBM, MD-300L, MEDA Co., Ltd., Tianjin, China).

### 2.3. Surgical Procedure

2% lidocaine and 1% ropivacaine were mixed and used to induce the retrobulbar anesthesia. Under the surgical microscope, four rectus muscles were tagged and the length of a 100 mm silicone encircling band (3 mm width, Beijing Jingcheng Medical Devices Co., Ltd, Beijing, China) was placed under the muscles. Subsequently, 25-gauge valved cannula (Alcon Laboratories Inc., Fort Worth, Texas, USA) was inserted into the vitreous cavity before subretinal fluid drainage. Retinal break identification and cryotherapy were performed under the direct visualization through chandelier illumination (Synergetics Inc., St Charles, Missouri, USA), microscope, and noncontact wide-angle viewing system (Carl Zeiss Meditec, Jena, Germany). Then, we removed chandelier illumination, marked retinal break, and tightened and sutured the encircling band. An appropriate size of element was placed on the surface of the sclera corresponding to retinal break. The location of the retinal break was finally confirmed by depressing element and viewing under chandelier illuminations. All surgeries were performed by the same surgeon.

## 3. Results

### 3.1. The Patient Demographics and Preoperative Findings Are Displayed in Table 1

Ten eyes of 10 patients aged 22 to 70 years were included (average age, 49.3 ± 18.9 years). There were six male subjects and four female subjects. Of 10 eyes, one eye (case 6) was hyperopic and nine were myopic, of which two eyes were high myopic (≥-6.00 DS). The average duration of RRD was 30.9 ± 53.3 days. Macula was involved in four eyes. The best corrected visual acuity before surgery ranged from 20/400 to 20/20.

### 3.2. Surgical Procedures and Postoperative Clinical Characteristics Are Displayed in Table 2

All ten eyes underwent surgical repair via 25-gauge chandelier illumination-assisted scleral buckling. Five eyes underwent 360-degree band with element surgery; five eyes underwent element surgery alone. The average length of the encircling band was 68.2 ± 1.3 mm. Element with widths of 7 mm and 9 mm was used; the average length was 10.5 ± 2.5 mm. 25-gauge chandelier illumination was usually placed in a quadrant opposite to the retinal breaks, eight eyes in superior nasal and two eyes in inferior temporal. The primary retinal reattachment was achieved in nine eyes. Due to the backward movement of the element, one patient (case 7) did not obtain complete retinal reattachment. Reattachment was achieved in case 7 after the second surgery by adjusting the position of element combined with intravitreal injection of sterile air. No intraoperative or postoperative complications were encountered in any of the 10 cases. At the last follow-up, nine eyes had a BCVA better than 20/40, four eyes achieved 20/20, and one eye was worse than 20/40 because of cataract. The mean follow-up period was 6.2 ± 0.9 years.

### 3.3. The Preoperative and Postoperative Result of Case 10 Is Shown in Figure 1 as an Example

**Figure 1:**
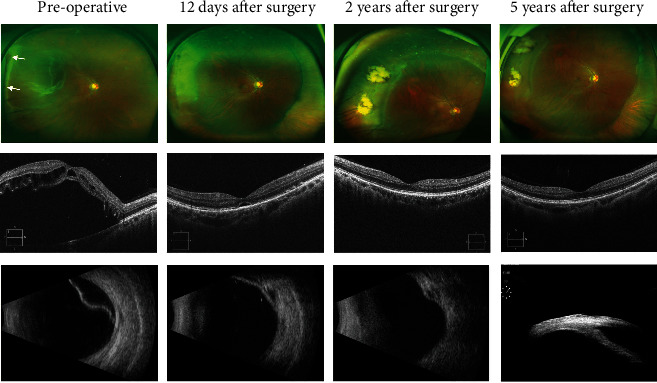
Examination result of case 10 before and after surgery. (a) Preoperative fundus photography showed retinal detachment and holes. (b–d) Fundus photography showed retinal reattachment, scleral buckle ridge, and cryoretinopexy scar at different times after surgery. (e) OCT demonstrated macular off and intraretinal cyst before surgery. (f–h) OCT showed macular reposition. (i) B-ultrasound confirmed retinal detachment. (j, k) B-ultrasound demonstrated retina reattachment and scleral buckle ridge. (l) 25-gauge chandelier insertion site was checked by UBM.

Before the surgery, fundus photography showed two retinal holes at both sides of the retinal lattice degeneration area, which spanned one clock (Figure 1(a)). OCT demonstrated that the macula was involved (Figure 1(e)) and B-ultrasound confirmed retinal detachment on the temporal side (Figure 1(i)). Fundus photography showed the reattachment of the retina, scleral buckle ridge, and cryoretinopexy scar at twelve days, two years, and five years after surgery, respectively (Figures 1(b)–1(d)). OCT showed gradual restoration of the macular structure (Figures 1(f)–1(h)). B-ultrasound also showed retina reattachment and scleral buckle ridge (Figures 1(j) and 1(k)). UBM examination of the 25-gauge chandelier insertion site on the pars plana revealed no tissue proliferation (Figure 1(l)).

## 4. Discussion

Scleral buckling is still the first-line option for treating some special RRD, like in young patients, and phakic and inferior break conditions [8]. A systematic review reported that scleral buckling provided superior postoperative outcomes than PPV on primary RRD [9]. Additionally, a recent large multicenter study confirmed better results (91.7%) of scleral buckling than PPV (83.1%) in moderately complex phakic primary RRD [10].

For making scleral buckling simplified and effective, a series of modifications from material, instrument, and surgical techniques have emerged. In 2012, Aras et al. [4] first reported modified scleral buckling using a noncontact wide-angle viewing system combined with a 25-gauge chandelier light source through an uncannulated sclerotomy for retinal visualization. Thereafter, different modified illuminations were tried and reported, such as 25-gauge chandelier light through a transscleral cannula [11], 25-gaugee “self-retaining endoilluminator” [12], twin uncannulated 27-gauge chandelier [13], and a guarded 25-gauge or 27-gauge light pipe [14]. Recently, Frisina et al. reported 213 eyes of primary RRD who underwent microscope-assisted ab externo surgery and demonstrated the safety and efficacy of this technique [15]. However, the reported studies on chandelier endoillumination-assisted scleral buckling cover a limited follow-up period, ranging from 1 to 12 months [3, 16–21]. Our group applied 25-gauge cannula-based chandelier-assisted scleral buckling since 2013. The average follow-up period of this study is up to 6 years. A longer follow-up of patients could help to further understand the complications related with chandelier endoillumination and better evaluate the recovery and stability of visual outcomes after surgery. None of the cases in this study have any occurrence of intraoperative and postoperative complications. Hu et al. [16] employed UBM to examine the incision of pars plana at 1 month and 3 months postoperatively, and no visible vitreous incarceration was found. In this study, UBM examination showed no tissue proliferation on the pars plana of 25-gauge chandelier insertion site. Except for the anatomical reposition of the retina, the development of cataracts is closely related to postoperative visual outcomes. Cataract development or progression is more prevalent in PPV, and PPV is associated with a slower visual recovery [22–24]. The average age of the patients in our study was 49.3 ± 18.9 years; six years after the surgery, 90% of patients had BCVA better than 20/40 and 40% of patients maintained 20/20. A recent multicenter study also confirmed that patients with phakic moderately complex primary RRD who underwent scleral buckling had significantly better visual outcomes [10]. Several studies have reported that the use of chandelier-assisted scleral buckling yielded a reattachment rate of 83.3-95.5% [3, 5]. The primary anatomical success rate in our study was 90%. Case 7 achieved retinal reattachment after the secondary surgery by adjusting the position of element. The limitation of our study is the small sample size, which may underestimate the success rate of surgery and intraoperative complications. Additionally, the absence of a control group was not sufficiently powered to statistically define the noninferiority of chandelier-assisted scleral buckling compared with conventional technique.

Recent update in chandelier-assisted scleral buckling includes using 3D visualization system [5, 14], antidrying contact lens [5], combination with an endolaser [25], and intravitreal injection of hyaluronate [26]. A new modification that continued to emerge may improve the ergonomics of surgeon and increase the attraction or popularity of chandelier-assisted scleral buckling.

In conclusion, one-size-fits-all technique does not exist in retinal detachment repair. Scleral buckling remains a very important surgical technique for the management of RRD. 25-gauge chandelier illumination and noncontact wide-angle viewing system-assisted scleral buckling are an effective and safe method for the treatment of selected RRD.

## Figures and Tables

**Table 1:** Basic clinical characteristics of the 10 cases.

Case	Age (y)	Sex	R/L	Diopter sphere	Course of disease (d)	Break location	Macular	Preop VA	Preop BCVA
1	65	Male	L	-0.25	20	3 o'clock	Off	20/80	20/80
2	22	Male	R	-7.00	180	8 o'clock	Off	20/400	20/400
3	64	Male	L	-5.00	7	2 o'clock	Off	20/80	20/50
4	31	Female	L	-3.00	3	2 o'clock	On	20/25	20/25
5	34	Male	L	-5.75	30	12:30 o'clock	On	20/63	20/20
6	66	Female	L	^+^2.75	14	10 o'clock	On	20/40	20/25
7	70	Male	R	-5.25	15	11 o'clock	On	20/200	20/50
8	68	Female	L	-4.00	30	12:30 o'clock	On	20/50	20/32
9	42	Female	L	-7.25	7	10 o'clock	On	20/100	20/20
10	31	Male	R	-5.50	3	9 o'clock and 10 o'clock	Off	20/63	20/63

VA: visual acuity; BCVA: best corrected visual acuity.

**Table 2:** Surgical procedures and postoperative clinical characteristics of the 10 cases.

Case	Surgical procedures	Length of band (mm)	Size of element^∗^ (mm)	Drainage	25G illumination	Complications	Retina	Postop BCVA	Follow-up period (y)	BCVA at last follow-up
1	360-degree band with element	69	12∗9∗3 mm	+	Superior nasal	None	Attached	20/100	5.5	20/25
2	360-degree band with element	66	8∗7∗3 mm	+	Superior nasal	None	Attached	20/400	7.3	20/32
3	Element alone		8∗7∗3 mm	+	Superior nasal	None	Attached	20/100	6.0	20/32
4	360-degree band with element	68	8∗7∗3 mm	+	Superior nasal	None	Attached	20/25	7.5	20/20
5	Element alone		8∗9∗3 mm	+	Superior nasal	None	Attached	20/20	5.8	20/20
6	Element alone		12∗7∗3 mm	+	Inferior temporal	None	Attached	20/50	5.2	20/20
7	360-degree band with element and sterile air	69	12∗9∗3 mm	+	Superior nasal	None	Not completely attached	20/50	6.8	20/25
8	Element alone		12∗9∗3 mm	+	Superior nasal	None	Attached	20/32	5.7	20/50
9	Element alone		10∗9∗3 mm	+	Inferior temporal	None	Attached	20/40	7.3	20/20
10	360-degree band with element	69	15∗9∗3 mm	+	Superior nasal	None	Attached	20/50	5.2	20/25

^∗^Element size described by length, width, and thickness; BCVA: best corrected visual acuity.

## Data Availability

The statistical clinical data used to support the findings of this study are included within the article.
