# Corrigendum to “Six-Year Outcomes of 25-Gauge Chandelier Illumination-Assisted Scleral Buckling”

**DOI:** 10.1155/2022/9842417

**Published:** 2022-01-06

**Authors:** Hui Li, Conghui Zhang, Jiayi Wei, Khusbu Keyal, Fang Wang

**Affiliations:** Department of Ophthalmology, Shanghai Tenth People's Hospital Affiliated to Tongji University, Shanghai 200072, China

In the article titled “Six-Year Outcomes of 25-Gauge Chandelier Illumination-Assisted Scleral Buckling” [1], there was an error in Figure 1, which should be corrected as follows:

Additionally, the acknowledgement section should be added as follows:

## Figures and Tables

**Figure 1:**
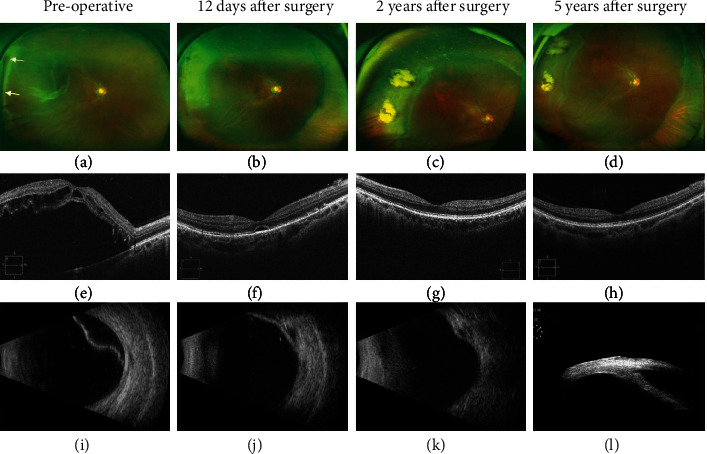
Examination result of case 10 before and after surgery. (a) Preoperative fundus photography showed retinal detachment and holes. (b)–(d) Fundus photography showed retinal reattachment, scleral buckle ridge, and cryoretinopexy scar at different times after surgery. (e) OCT demonstrated macular off and intraretinal cyst before surgery. (f)–(h) OCT showed macular reposition. (i) B-ultrasound confirmed retinal detachment. (j, k) B-ultrasound demonstrated retina reattachment and scleral buckle ridge. (l) 25-gauge chandelier insertion site was checked by UBM.
